# Anxiety among dental professionals and its association with their dependency on social media for health information: insights from the COVID-19 pandemic

**DOI:** 10.1186/s40359-020-00509-y

**Published:** 2021-01-21

**Authors:** Suhail H. Al-Amad, Amal Hussein

**Affiliations:** 1 Department of Oral and Craniofacial Health Sciences, Room M28-132, College of Dental Medicine, University of Sharjah, Sharjah, United Arab Emirates; 2 Department of Family and Community Medicine and Behavioural Sciences, College of Medicine, University of Sharjah, Sharjah, United Arab Emirates

**Keywords:** Anxiety, Social media, Dentists

## Abstract

**Background:**

Social media can play a detrimental role during a global health emergency. In this study, we aimed at assessing the impact social media has on the anxiety level of dental healthcare workers (DHCWs) whilst living through the COVID-19 pandemic.

**Methods:**

An online questionnaire was disseminated to a cross-sectional sample of DHCWs from 19 countries using social media platforms. The questionnaire enquired about DHCWs’ frequency of using social media and their dependency on health-related information posted on those platforms. Anxiety was measured using General Anxiety Disorder scale (GAD-7).

**Results:**

Four-hundred and three (403) DHCWs completed the online questionnaire. Sixty-eight percent (68%) frequently use social media for information on COVID-19. The frequency of social media use was higher among younger DHCWs, with shorter clinical experience, and holders of undergraduate qualifications (*p* = 0.009, *p* = 0.002, and *p* = 0.023, respectively). Almost one third of DHCWs had moderate to severe anxiety (31.7%), which was significantly associated with the frequency of social media use (*p* = 0.016). This association was adjusted for age, years of experience and qualification level (OR 1.75; 95% CI 1.05–2.93; *p* = 0.032).

**Conclusion:**

COVID-19 social media infodemic has been adversely impacting the psychological wellbeing of DHCWs. More effective measures are needed to control the quality and spreadability of health information on social media platforms.

## Background

Since the early days of its identification, COVID-19 has been exponentially trending on social media platforms in an unprecedented manner. Posts on the viral origin, its pathogenesis and transmissibility have flooded social media platforms worldwide in a new phenomenon that became known as “infodemic” [1].

The non-specific clinical manifestations, the uncertainties around the viral transmissibility, and the unexplainable wide range of mortality rate [2–4] were among the factors that created knowledge voids, which were quickly filled with often scientifically unfounded information on social media platforms. Unlike previous disease outbreaks, health authorities around the world found themselves compelled to contain a COVID-19 infodemic, along their conventional measures to contain the viral pandemic. The easy-to-use technology-driven phenomenon has been causing confusion and uncertainties about COVID-19 pandemic among laypersons and healthcare providers alike.

Dental professionals are at the highest risk of contracting SARS-CoV-2 [5]. This can be attributed to a number of factors: firstly, the ergonomics of dental treatments that require dental healthcare workers (DHCWs) to be at a close proximity to the patient’s oral cavity, and for extended periods of time. Secondly, most dental treatments generate large volume of aerosols, which eventually land on the clinician’s face, head and neck [6]. Thirdly, saliva has been found to be a rich source of SARS-CoV-2 [7]. And fourthly, viral particles in aerosols were found to remain viable for as long as three hours [8].

Concerns over the psychological wellbeing of healthcare workers and laypersons have been raised [9–12]. Social media infodemic has been linked to depression and anxiety among a large cross-sectional sample of Chinese laypersons [10]. It is not clear if dental professionals, who are at a substantial occupational risk of acquiring COVID-19, are psychologically vulnerable to social media health-related infodemic.

In this study, we aimed at investigating the DHCWs’ frequency of social media use, their dependency on the COVID-19 pandemic’s health-related information posted on those platforms, and whether this dependency has an association with dental professionals’ psychological wellbeing. We hypothesize that a higher frequency of using social media infodemic is associated with a higher anxiety level among DHCWs.

## Methods

This research has been independently reviewed and approved by the University of Sharjah Research Ethics Committee (approval No: REC-20-04-04-02). The said committee works in accordance with the ethical standards of the 1964 Declaration of Helsinki and its later amendments.

### Design and sample

This was a cross-sectional study which was conducted through an online survey. An invitation, which included a link to an online survey, was disseminated by the authors to DHCWs using Emails, WhatsApp and Facebook messaging platforms. Using a snowball sampling method [13], recipients were asked to use their social media networks to forward this invitation to their acquaintances of dental healthcare providers regardless of the country where they are practicing. The targeted population were dentists, dental specialists, dental hygienists, dental nurses and dental technicians who were actively practicing dentistry at the time of the study. Sample size was calculated using the following formula: N = {1.96^2^ × p × (1 − p)}/ME^2^; where p was set at 50% and margin of error at 5%. For a 95% confidence level, the minimum sample size needed was 385.

## Research tool

An online questionnaire using Google forms was specifically designed for this study (see Additional file 1). The online questionnaire was pilot tested for clarity and coherence and was modified based on the received feedback. The questionnaire included three sections as follows: Section A enquired about basic demographic variables. Those included the age, sex, years of clinical experience, dental professional category, participants’ highest qualification, practice sector and country of practice.

Section B enquired about the frequency of using social media platforms for information on COVID-19 during the previous 14 days. In addition, participants were asked about the frequency of them actively verifying the information that they receive on COVID-19 through social media, and their tendency to visit official websites and read journal articles to learn more about the transmissibility of COVID-19 in dentistry. Answers of this section were registered on a 4- or 5-Likert scale [14].

Finally, section C assessed the level of anxiety using the 7-item Generalized Anxiety Disorders scale (GAD-7). Participants were asked to rank their feelings over the past 14 days towards seven anxiety-related statements using a scale that ranged from “not at all” to “nearly every day”. Anxiety was categorised into minimal, mild, moderate and severe based on the overall cumulative scores [15].

### Statistical analysis

IBM® SPSS® Statistics (version 26) (IBM Corporation) was used for statistical analysis. Age and clinical experience were categorized into three year-range categories. Values presented in Likert scale were clustered into two or three categories to minimize the number of cells with expected counts less than 5. Internal consistency reliability of the GAD-7 scale was measured using Cronbach’s Alpha. Demographic variables were summarized in frequencies and percentages, and Chi-square test was used to assess the associations between various categorical variables. Binary Logistic Regression models were used to identify predictors of frequent use of social media and moderate/severe anxiety group. p-value was considered significant if < 0.05.

## Results

Four-hundred and three (403) dental healthcare workers (DHCWs) participated in this online survey. The mean age of participants was 36.3 years (SD = 9.7), ranging from 23 to 75 years. Females represented 70% of the sample. The mean duration of clinical experience was 12.3 years (SD = 9.6).

Most of the participating DHCWs were dentists (n = 245 (60.8%)), who were less than 40 years of age (n = 274 (69.2%)) and working in the private sector (n = 179 (44.4%)). DHCWs were practicing dentistry in 19 different countries, those were (in alphabetical order): Bahrain, Canada, Egypt, Germany, India, Italy, Jordan, Kuwait, Malaysia, Oman, Palestine, Poland, Qatar, Saudi Arabia, Syria, Turkey, United Arab Emirates, United Kingdom, United States of America. The majority of participants were working in a Middle Eastern country (n = 370 (91.8%)) (Table 1).

**Table 1:** Description of study sample by socio-demographics characteristics and frequency of using internet resources

Variable		N (%)
Age	= < 30 years	140 (35.4)
	31–40 years	134 (33.8)
	> 40 years	122 (30.8)
Sex	Males	120 (29.9)
	Females	282 (70.1)
Clinical experience	1–5 years	131 (32.9)
	6–15 years	132 (33.2)
	> 15 years	135 (34.0)
Professional category	Dentist	245 (60.8)
	Dental specialist	123 (30.5)
	Dental auxiliary^a^	34 (8.4)
Qualification level	Undergraduate	218 (54.1)
	Postgraduate	183 (45.4)
Practice sector	Private clinic	179 (44.4)
	Government clinic	86 (21.3)
	University teaching clinic	96 (23.8)
	Combined	36 (8.9)
Jurisdiction region	Middle East	370 (91.8)
	Non-Middle East	29 (7.2)
Dental specialty	Periodontics	14 (9.3)
	Prosthodontics	25 (16.6)
	Restorative dentistry	11 (7.3)
	Endodontics	26 (17.2)
	Oral and oral and maxillofacial surgery	17 (11.3)
	Orthodontics	29 (19.2)
	Pediatric dentistry	28 (18.5)
	Others	1 (0.7)
Frequency of using social media	Frequently	274 (68.0)
	Infrequently	129 (32.0)
Frequency of visiting official public health websites^b^	Frequently	316 (79.0%)
	Infrequently	84 (21.0%)
Frequency of reading scientific journals	Frequently	177 (44.7%)
	Infrequently	219 (55.3%)

^a^Includes dental interns, dental hygienists, dental nurses and dental technicians

^b^World Health Organization, Centre for Disease Control and Prevention, national health jurisdictions

All participants in our sample had an account in at least one social media platform. The most commonly used platform was WhatsApp (100%), followed by Instagram and Facebook (98% for each), while the least used platform was Tiktok (87%).

Overall, 68% of the sample considered themselves to be frequent users of social media for information on COVID-19. WhatsApp and Instagram were the most commonly used platforms for COVID-19 information (41% and 39.6%, respectively), while the least used ones were Snapchat and Tiktok (7.6% and 1.1%, respectively).

Only 7.4% of our sample stated that they frequently write—by themselves—posts related to information on COVID-19, while 69.3% stated that they frequently verify the accuracy of the information that they receive on social media. Around one third of participants (32.3%) stated that most of the information they received on their social media turned out -at a later time- to be a rumour.

**Fig. 1:**
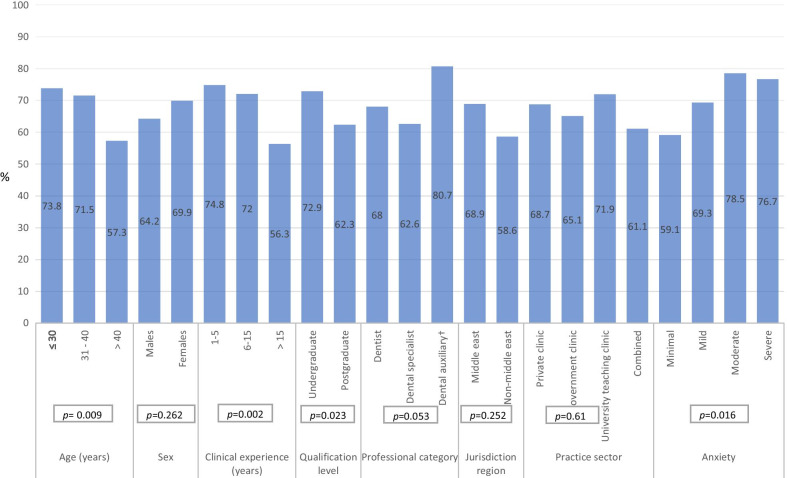
Bivariate analysis showing associations between the frequency of using social media for information on COVID-19 and various socio-demographic factors. Includes dental interns, dental hygienists, dental nurses and dental technicians

The frequency of utilizing social media for COVID-19 information was significantly associated the DHCWs’ age (*p* = 0.009), their years of clinical experience (*p* = 0.002) and their qualification level (*p* = 0.023), whereby older generations and postgraduate degree holders were less frequent users (Fig. 1).

**Fig. 2:**
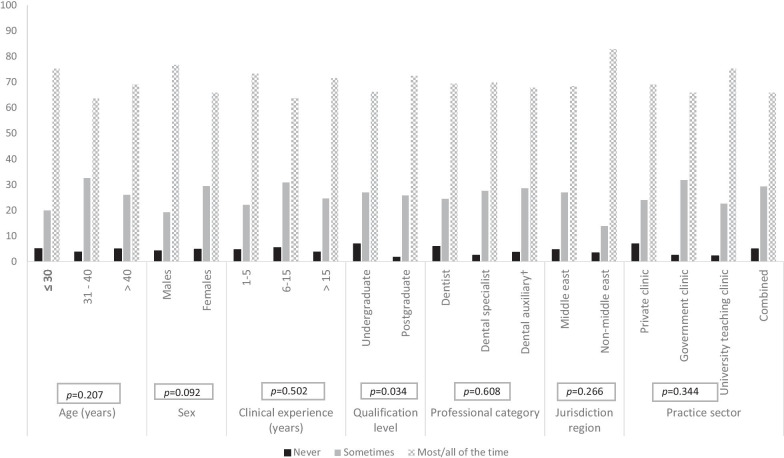
Bivariate analysis showing associations between the tendency to verify COVID-19 information on social media and various socio-demographic factors. Includes dental interns, dental hygienists, dental nurses and dental technicians

With regards to the perceived reliability, the majority of DHCWs considered the information on COVID-19 which were presented through television news channels and infographic social media posts as reliable (86.9% and 56.5%, respectively), while the least reliable source was social media posts consisting of plain text (35.5%). Regardless of their demographic status, most DHCWs expressed a tendency to frequently verify the information on COVID-19 that they receive on social media before accepting it. However, the ones with the greatest tendency to do so were DHCWs with postgraduate qualifications (*p* = 0.034) (Fig. 2).

Reliability testing of GAD-7 scale revealed a Cronbach’s Alpha of 0.937. Almost one third (31.7%) of DHCWs in our sample scored values that were indicative of moderate or severe anxiety based on the GAD-7 scale. Females and those who are more frequent users of social media showed higher levels of anxiety (*p* < 0.0005 and *p* = 0.016, respectively) (Table 2). The associations between moderate/severe anxiety and female gender (OR 2.01; 95% CI 1.15–3.49; *p* = 0.014) and between anxiety and frequency of social media use (OR 1.75; 95% CI 1.05–2.93; *p* = 0.032) were independent of age, years of experience, and professional category (Table 3).

**Table 2:** Association between general anxiety and various socio-demographic variables

Variable	Categories	Total N (%)	Anxiety groups based on the GAD scores				*P*-value
			Minimal (score between 0–4) N (%)	Mild (score between 5–9) N (%)	Moderate (score between 10–14) N (%)	Severe (score between 15–21) N (%)	
Age	= < 30 years	139 (35.4)	49 (35.3)	48 (34.5)	21 (15.1)	21 (15.1)	0.096
	31–40 years	135 (34.4)	37 (27.4)	49 (36.3)	20 (14.8)	29 (21.5)	
	> 40 years	119 (30.3)	46 (38.7)	40 (33.6)	23 (19.3)	10 (8.4)	
Sex	Males	116 (29.5)	58 (50.0)	35 (30.2)	13 (11.2)	10 (8.6)	< 0.0005
	Females	277 (70.5)	74 (26.7)	101 (36.5)	52 (18.8)	50 (18.1)	
Clinical experience	1–5 years	129 (33.2)	48 (37.2)	45 (34.9)	20 (15.5)	16 (12.4)	0.074
	6–15 years	131 (33.7)	35 (26.7)	43 (32.8)	24 (18.3)	29 (22.1)	
	> 15 years	129 (33.2)	49 (38.0)	49 (38.0)	18 (14.0)	13 (10.1)	
Qualification level	Undergraduate	216 (55.1)	73 (33.8)	76 (35.2)	32 (14.8)	35 (16.2)	0.750
	Postgraduate	176 (44.9)	58 (33.0)	60 (34.1)	33 (18.8)	25 (14.2)	
Professional category	Dentist	218 (55.5)	78 (35.8)	76 (34.9)	33 (15.1)	31 (14.2)	0.063
	Dental specialist	118 (30.0)	44 (37.3)	42 (35.6)	16 (13.6)	16 (13.6)	
	Dental auxiliary^a^	57 (14.5)	10 (17.5)	19 (33.3)	15 (26.3)	13 (22.8)	
Jurisdiction region	Middle east	361 (92.6)	120 (33.2)	126 (34.9)	61 (16.9)	54 (15.0)	0.967
	Non-middle east	29 (7.4)	10 (34.5)	11 (37.9)	4 (13.8)	4 (13.8)	
Practice sector	Private clinic	175 (45.1)	69 (39.4)	54 (30.9)	33 (18.9)	19 (19.9)	0.132
	Government clinic	84 (21.6)	27 (32.1)	34 (40.5)	8 (9.5)	15 (17.9)	
	University teaching clinic	93 (24.0)	26 (28.0)	33 (35.5)	15 (16.1)	19 (20.4)	
	Combined	36 (9.3)	8 (22.2)	15 (41.7)	7 (19.4)	6 (16.7)	
Use of social media for information on COVID-19	Infrequently	124 (31.5)	54 (43.5)	42 (33.9)	14 (11.3)	14 (11.3)	0.016
	Frequently	270 (68.5)	78 (28.9)	95 (35.2)	51 (18.9)	46 (17.0)	

^a^Includes dental interns, dental hygienists, dental nurses and dental technicians

**Table 3:** Binary Logistic Regression models identifying predictors of frequent use of social media and Moderate/Severe Anxiety group

	β	se	*p*-value	Adjusted OR (Exp β)	95% Confidence Interval	
					Lower	Upper
Predictors of frequent use of social media						
Age						
≤ 30	− 0.392	0.685	0.567	0.676	0.176	2.589
31–40	0.027	0.424	0.950	1.027	0.448	2.356
> 40	–	–	–	1 (Reference)	–	–
Level of qualification						
Undergraduate	0.498	0.343	0.146	1.646	0.840	3.223
Postgraduate	–	–	–	1 (Reference)	–	–
Experience in dental practice						
1–5	0.934	0.686	0.173	2.545	0.663	9.766
6–15	0.376	0.435	0.388	1.457	0.621	3.421
> 15	–	–	–	1 (Reference)	–	–
Dental professional						
Dentist	–	–	–	1 (Reference)	–	–
Dental specialist	0.354	0.352	0.314	1.425	0.715	2.840
Dental auxiliary^a^	0.302	0.388	0.437	1.353	0.632	2.896
Anxiety group						
Minimal	–	–	–	1 (Reference)	–	–
Mild	0.451	0.265	0.088	1.570	0.935	2.637
Moderate/Severe	0.836	0.295	0.005	2.307	1.295	4.110
− 2LogLikelihood = 459.361 *χ*^2^ (df = 9) = 19.954 *p* value = 0.018						
Predictors of moderate/severe anxiety group						
Age						
≤ 30	–	–	–	1 (Reference)	–	–
31–40	− 0.050	0.534	0.926	0.951	0.334	2.709
> 40	0.339	0.675	0.615	1.404	0.374	5.266
Sex						
Male	–	–	–	1 (Reference)	–	–
Female	0.696	0.282	0.014	2.005	1.153	3.486
Experience in dental practice						
1–5	–	–	–	1 (Reference)	–	–
6–15	0.587	0.530	0.268	1.798	0.637	5.076
> 15	− 0.101	0.687	0.883	0.904	0.235	3.476
Dental professional						
Dentist	–	–	–	1 (Reference)	–	–
Dental specialist	− 0.019	0.289	0.948	0.981	0.557	1.727
Dental auxiliary	0.545	0.326	0.095	1.724	0.910	3.267
Use of social media						
Infrequently	–	–	–	1 (Reference)	–	–
Frequently	0.562	0.262	0.032	1.754	1.049	2.932
− 2LogLikelihood = 450.102 *χ*^2^ (*df* = 8) = 25.303 *p* value = 0.001						

^a^Includes dental interns, dental hygienists, dental nurses and dental technicians

## Discussion

Today, 51% of the world population are users of the internet [16]. Information of diverse nature and quality, including health information, are being disseminated between people at a scale that is wider and faster than ever before. In a large study which included 42,087 participants, Din et al. reported that more than half their sample used the internet to obtain information related to health, the majority of whom were young, females and of a higher socioeconomic status [17].

Social media platforms are increasingly becoming popular by people of all ages, ethnicities and socioeconomic backgrounds. In the United States—for example—percentage of social media users jumped from 5% in 2005 to 72% in 2019 [18]. The International Telecommunication Union estimates that in 2019, 46% of the world’s population were active users of social media platforms [16]. Healthcare providers are likewise increasingly engaged in social media use. A recent survey showed that around 88% of healthcare workers have social media accounts that they use on daily basis [19, 20].

The rapid pace of news and uncertainties around the viral genesis, transmissibility and pathogenesis led to information voids which were conveniently and quickly filled with huge amount of social media posts, in a new phenomenon that became known as “infodemic” [1, 19–22]. The rapid spread of information, some of which were later discredited, has floundered people and created a wide-spread anxiety [23, 24].

While most governments have imposed strict measures to reduce the impact of COVID-19 on healthcare infrastructure, the psychological impact of this viral pandemic was not addressed with an equivalent magnitude. As a result, anxiety has been a morbidity rapidly emerging in the background for both laypersons and healthcare professionals, among whom are dental healthcare workers (DHCWs).

Based on the O*NET database, the calculated risk of contracting SARS-CoV-2 is greatest for dentists [5]. With this in consideration, we aimed at investigating the frequency of DHCWs’ use of social media for information on COVID-19, and the association between this frequency and DHCWs’ psychological wellbeing. Additionally, we aimed at assessing DHCWs’ tendency to verify the correctness of COVID-19 information which they receive.

Our results revealed a high prevalence of social media use among DHCWs, whereby all participants have an account in at least one social media platform. More than two-thirds of them frequently rely on social media for information on COVID-19. The frequency of using social media was significantly associated with younger age, shorter clinical experience and lower academic qualifications, which can be explained by the fact that social media and smart hand-held devices are modern-day trends, to which younger generations would be more accustomed to.

While television news channels were perceived as the most reliable source of COVID-19 information, social media posts with infographics had a greater influence on the perceived reliability by comparison with posts consisting of plain text alone. Infographics have been shown to be effective tools in disseminating knowledge, including health information, to audiences from various backgrounds [25–27].

Social media has been playing an increasingly powerful role in disseminating health information. However, when wrong health information was received by desperate persons, serious health consequences ensued [28]. Health information on social media platforms were found to have serious misconceptions [28–30]. Healthcare workers are under the obligation to verify the correctness of health-related information when they receive them. Comfortingly, more than two thirds of our sample indicated that they do verify the accuracy of information which they receive via social media before accepting it. This tendency was significantly associated with the dental professionals who had postgraduate qualifications.

To assess anxiety among DHCWs, we used GAD-7 scale [15], which consists of a simple 7-item questionnaire that assesses the level of anxiety based on a cumulative score. The tool has been validated and found to be a reliable and valid tool to measure anxiety among the general population [31], as well as healthcare providers [32]. In our study, GAD-7 scale showed high reliability among dental practitioners, as indicated by Cronbach’s Alpha test (0.937).

Almost one third of our sample gave a cumulative GAD-7 score that ranged from 10 to 21, indicating a state of moderate to severe anxiety. Females and frequent users of social media were two variables significantly associated with higher anxiety levels (*p* < 0.0005 and *p* = 0.016, respectively), and this association was independent of age, years of clinical experience and professional category.

Awareness to healthcare workers’ mental health has received emphasis in the countries that suffered from COVID-19 at an early stage [9, 11, 12]. Gau et al. reported that 82% of the general population in China were frequent users of social media and the prevalence rate of anxiety among that population was 22.6% with a significant association between the two [10].

The difference in the rates of both social media use and anxiety between our study and that of Gau et al. can be attributed to the sample selection and sample size. Whilst Gau et al. conducted their survey on laypersons who were as young as 18 years [10], our sample consisted of older and dentally qualified individuals. Despite the difference in frequencies, both studies present an interesting and significant association between anxiety and the frequency of social media use, which strengthens the inference that social media plays a negative role when it is used as a source of health information, particularly amid a global health emergency.

## Conclusion

In this study, we showed a relatively high prevalence of social media use among DHCWs, which was significantly associated with their anxiety level. The growing emphasis on mental health, particularly among healthcare workers, should take into account its predisposing factors, one of which is modern-day social media infodemic. Despite this finding, the cross-sectional design of our study is not ideal to prove a causal relationship between anxiety and the frequency of social media use, which we observe as a limitation to the generalization of this particular finding.

As dental clinical services gradually resume, we recommend that health jurisdictions pay more attention to the mental wellbeing of those who are most exposed to COVID-19, including DHCWs. This should include regular debriefing and counselling sessions. Access to mental healthcare should also be made available. While some form of filtration of scientific information on social media should be put in place, healthcare providers in general should be discouraged from relying on social media for updates.

## Supplementary information

**Additional file 1.**

## Data Availability

Data is available upon request from the corresponding author.

## Abbreviations

DHCWs: Dental Healthcare Workers
GAD-7: General Anxiety Disorder-7 scale
SARS-CoV-2: Severe Acute Respiratory Syndrome Coronavirus 2
