# Supplementary material for: Anxiety among dental professionals and its association with their dependency on social media for health information: insights from the COVID-19 pandemic
Source: BMC Psychol. 2021 Jan 21;9:9. doi: 10.1186/s40359-020-00509-y (PMC7819620; doi:10.1186/s40359-020-00509-y)
Supplement: Supplementary file 1 — Additional file 1. [file 40359_2020_509_MOESM1_ESM.docx]

**COVID-19 ‘infodemic’ and its impact on routine practice by dental professionals**

Dear dental healthcare provider,

We are a group of researchers from the **University of Sharjah** conducting a questionnaire-based study on the impact of information on social media on your routine dental practice, amid the surg of COVID-19 information that is taking place worldwide.

Our questionnaire is divided into **three** sections, as follows:

- **Section A** will enquire about basic non-identifying demographic data
- **Section B** will enquire about your personal use of social media
- **Section C** will enquire about the level of anxiety that you might be having amid the COVID-19 pandemic.

Filling this questionnaire will take no more than 20 minutes of your time. We thank you for sparing your valuable time to do so.

Our questions are not of sensitive nature. Your answers will be completely anonymous.

You are of course under no obligation to participate. But we do encourage you to do so as we feel that your answers will generate useful results for all interested persons.

If you have any questions regarding this study, please call or write to the Principle Investigator of this study **Dr Suhail Al-Amad** ([salamad@sharjah.ac.ae](mailto:salamad@sharjah.ac.ae)), telephone: 0507365702.

If you have concerns or complaints about this study, please write to the **University of Sharjah Research Ethics Committee** at [rec@sharjah.ac.ae](mailto:rec@sharjah.ac.ae)

We sincerely thank you for your cooperation with us in this research.

**Section A: Demographic data**

**Tell us a little bit about yourself**

1. What is your age?

________

1. How many years have you been practicing dentistry?

_______

1. What is your sex?

- Male
- Female

1. Do you have children below 16 years who are living with you in the same house?

- Yes
- No

1. Do you have elderly above 70 years who are living with you in the same house?

- Yes
- No

1. What your highest qualification?

- Diploma
- Bachelor
- Higher Diploma
- Master
- PhD
- Clinical doctorate
- Professional specialization degrees, such as fellowships and board certificates

1. Are you a

- Dentist
- Dental specialist
- Dental hygienist
- Dental nurse
- Dental technician
- Dental intern

1. Are you working in a

- Private clinic
- Government clinic
- University teaching clinic
- Others

1. Which country are you currently working in?

______

1. If you are a dental specialist, what is your specialty

- Periodontics
- Prosthodontics
- Restorative/conservative dentistry
- Endodontics
- Oral surgery, or oral and maxillofacial surgery
- Orthodontics
- Paediatric dentistry
- Oral medicine, or oral pathology, or oral radiology
- Others

1. Before the COVID-19 outbreak, what was the approximate number of patient that you were seeing on a routine working day?

**______**

1. Are ROUTINE (NON-EMERGENCY) dental services currently suspended in your health jurisdiction as a result of the COVID-19 pandemic?

- Yes
- No

**Section B: Your engagement in social media**

**Here, we would like to ask you questions related to your reliance on social media for information about COVID-19**

1. How much have you been using the following social media to get your information related to COVID-19?

|  | Never | Rarely | Sometimes | Often | Always | I don’t have an account |
| --- | --- | --- | --- | --- | --- | --- |
| Facebook |  |  |  |  |  |  |
| Instagram |  |  |  |  |  |  |
| WhatsApp |  |  |  |  |  |  |
| Twitter |  |  |  |  |  |  |
| Snapchat |  |  |  |  |  |  |
| Tiktok |  |  |  |  |  |  |

1. Please rate the following resources in terms of their reliability for health-related information on COVID-19?

|  | Not reliable at all | Hardly reliable | Somewhat reliable | Very reliable |
| --- | --- | --- | --- | --- |
| Television news reports by News channels |  |  |  |  |
| Television news reports by Entertainment channels |  |  |  |  |
| Social media messages with infographics or videos |  |  |  |  |
| Social media messages with plain text |  |  |  |  |

1. During the past 14 days, how often did you ‘like’ or ‘share’ social media posts related to:

|  | Never | Rarely | Sometimes | Often | Always |
| --- | --- | --- | --- | --- | --- |
| COVID-19 incidence and death rates |  |  |  |  |  |
| News reports on medicinal therapies or vaccines for COVID-19 |  |  |  |  |  |
| News reports on herbal therapies for COVID-19 |  |  |  |  |  |
| News reports on the transmissibility of the virus |  |  |  |  |  |
| Journal research papers on COVID-19 |  |  |  |  |  |

1. During the past 14 days, how often did you write posts yourself about COVID-19

- Never
- Few times
- Several times
- Everyday

1. During the past 14 days, how much information did you receive on COVID-19 through social media turned out to be a rumour?

- None of the information
- Some of the information
- Most of the information
- All of the information

1. How often do you verify the accuracy of information which you receive on social media, before accepting the information and/or sharing it?

- I never verify
- Sometimes I verify
- Most of the time I verify
- I verify all the time

1. When you go to sleep, how far (on average) will your smart phone be away from your head?

- < 0.5 meter from me
- 0.5 – 1.0 meter
- Between 1-2 meters
- > 2.0 meters
- My smart phone will be in a another room
- I don’t have a smart phone

1. How often do you check your social media account(s) upon waking up, and while you are still in bed?

- Never
- Only a day or two in a week
- Often
- Every morning

1. During the previous 14 days, how often have you visited any of the following websites to learn more about COVID-19 transmissibility in dentistry?

| Website | Never | Rarely | Frequently | Everyday |
| --- | --- | --- | --- | --- |
| Centres for Disease Control  [www.cdc.gov](http://www.cdc.gov) |  |  |  |  |
| World Health Organization  [www.who.int](http://www.who.int) |  |  |  |  |
| The health jurisdiction where you are licensed |  |  |  |  |
| Scientific journals |  |  |  |  |

**Section C: This final section is to assess your general anxiety.**

Please answer the questions below with the COVID-19 pandemic era in your mind.

1. Over the past 14 days, how often have you been bothered by the following?

|  | **Not at all** | **Several days** | **More than half the days** | **Nearly every day** |
| --- | --- | --- | --- | --- |
| Over the past 14 days, I was feeling nervous, anxious, or on edge |  |  |  |  |
| Over the past 14 days, I was not able to stop or control worrying |  |  |  |  |
| Over the past 14 days, I was worrying too much about different things |  |  |  |  |
| Over the past 14 days, I had trouble relaxing |  |  |  |  |
| Over the past 14 days, I was being so restless that it is hard to sit still |  |  |  |  |
| Over the past 14 days, I was becoming easily annoyed or irritable |  |  |  |  |
| Over the past 14 days, I was feeling afraid as if something awful might happen |  |  |  |  |

Thank you for sparing time to participate in our survey
